# Class-Room Talks No. 5

**Published:** 1888-06

**Authors:** 


### CLASS-ROOM TALKS. No. 5.

(From Lectures by Professor J. T. Kent, A. M., M. D.)

I am again reminded to caution you against the hasty use of such deep-acting remedies as Sulph. and Sil. when you have reason to suspect a chronic miasm has had expression in the system, without inquiring carefully into the history of that earlier expression, as such prescribing may give rise to dangerous complications.

I saw a case in which Sulphur had been perfectly indicated, but on being given too late, caused the patient to die shortly. Why? The history of the case had not been fully taken, or it would have shown that the patient, an old lady, had an attack of apoplexy about five years before. The result of the prescription showed that the clot, instead of resorbing, had become encysted; that Sulph. had set up the inflammatory process, leading to suppuration and death. Short acting remedies only should be given in such cases.

In tuberculosis, if the patient is known to have passive tubercles in the lungs, a dose of Sulph. or Sil. may set up so extensive a suppuration as to cause immediate death. Remember, then, in cases where there is possibility of encysted foreign bodies, especially among the deeper and more vital organs, to leave such remedies as Sulph. and Sil. out of the question.

In cases of rheumatic stiffness, with morning *aggravation*, Phosphorus leads all other remedies. A curious case presented, in which a lady friend asked my help. She had a dog, old and faithful, to whom she had been greatly attached because of his usefulness and fidelity. He had become so old and stiff he was of very little comfort to himself or to any one else. She asked one day that I prescribe for him. Well, the dog could hardly *tell* symptoms, but he *had* them. Upon inquiry I learned that he was so *stiff in the morning* he could hardly walk or move, and that it took him hours to become limber enough to get about a *little bit*. I wondered a little as to what I could give *a dog*, but because of the *aggravation* in the morning tried a dose of Phos., a high potency, and to my great surprise the fellow became a very comfortable old dog, running about with ease for a year or two afterward. Phos. is a very frequently indicated remedy for this stiffness coming on in old age.

In wasting disease, as consumption in some of its forms, last stages, say five or six weeks prior to death, the patient is beginning to suffer greatly ; hectic fever every afternoon ; violent, prolonged, circumscribed red spots upon the cheeks ; the last stage of suppuration ; profuse night-sweats ; thirst for ice-cold water. What will you do? Phos. is a dangerous remedy in these conditions if repeated. You will give it, of course—*one dose, very high*—as you value the life intrusted to you. Now wait. Within a day or two you may be sent for in great haste. The patient is suddenly worse. There is profuse, watery, offensive diarrhœa, gushing from her like a hydrant ; wide open anus ; fæces passing without her knowledge. What will you do now? Sac. Lac., of course. The diarrhœa will abate itself within a day, and your patient will become comfortable, live a few weeks, and pass away without further suffering. You *must* relieve these violent hectic conditions, and if you know nothing to give but Morphia, you will have the dissatisfaction of seeing them end their days in most horrible suffering, as there comes a time when Morphine can help no longer, after which the suffering is terrible to witness.

Later, another condition may arise ; the sensation of sinking, of dying, with cold breath, cold sweat, cold body ; a friend stands on either side of the couch gently *fanning the patient ;* it is seemingly the only way he can get his breath, the exhaustion is so great ; here is the Hippocratic countenance with blueness of the face, sunken mouth and nose. Carb. veg. is indicated and will give the patient rest and comfort for another space—perhaps a week or two.

Again, it may be a sensation of a heavy load upon the chest ; a horrible sense of suffocation ; a clutching and a palpitation of the heart ; a throwing off of the clothes or covering ; a tearing of the neck and chest ; continual swallowing and choking ; horrible suffering. Lachesis will relieve and the closing hours are made comparatively easy.

Another time you will find the patient in horrible restlessness and suffering. The body is tossing from side to side. The agony and prayers for death are insupportable; the pains extend over the whole body; the stinging and pricking are intense. The pain is not one great pain extending over the body, but the numerous little tormenting pains, the many in one, pricking, stinging, like so many hot needles (Ars. has served well), and telling to the intelligent physician of the death of the cells. Now, when one dose of Tarent. will ease this suffering, allowing the patient to die perfectly happy and comfortable within a day or two, would you give Morphia?

Phosphorus has an aggravation from lying upon the left side, like Nat-m.; but do not confuse the *conditions* which are made worse by lying upon the left side. In Nat-m. it is the heart symptoms, the palpitations, conditions brought about by disease of that organ; in Phosphorus it is the chest symptoms, the diseased condition of the lungs, the pain, the cough, stitches, dyspnœa. The cough will be made worse, even if it is a cough caused by tickling in the throat-pit, or it may be a symptom caused by the soreness, the rawness, the weakness, and consequent trembling. All chest symptoms will be *aggravated* by lying upon the left side. You will find in the repertories Nat-m. with an *aggravation* from lying upon the left side, and Merc. *aggravated* by lying upon the right side. We easily comprehend the symptom when we remember that Merc. has an especially broad sphere of action in chronic disease of the liver, where there is inflammation, enlargement, and induration of that organ, which it both produces and cures, and in which conditions we find distress, tenderness, and discomfort from pressure upon the organ, or, as the *Materia Medica* has it, *aggravated* from lying upon the right side.

Many of you who begin your practice or *continue* it in this portion of the United States, or in the adjacent States, will have patients coming up to you from the malarial districts farther south, maybe Arkansas. There people have lived in the atmosphere of the swamps of that region, breathing the foulness emanating from the decayed vegetable matters until they are thoroughly impregnated with the poison. Every little while Dame Nature has tried to expel these poisons, as is evidenced by the ague; "chills;" and these "chills" have been suppressed with Quinine. They have reached a point of emaciation and sallowness that looks cadaverous. They have developed through this suppression a chronic diarrhœa or chronic hepatitis, and are jaundiced, melancholic, and infirm.

When you have a case of that kind you will be glad to have known the power of Nat-m. to produce these conditions in the prover. It is a condition of starvation, inanition, for common salt. Nat-m. has just that depressing effect upon the vital forces of man, destroying the tonicity of organs and vessels, and creating the functional disturbances found present. This disorganization or depression of animal functions is shown in the symptoms: "late learning to talk;" "inability to think or to reason clearly," frequently seen in school-boys, who may look well, comparatively robust physically, fleshy, yet they will be clumsy, stupid at their books, excitable, irascible; canine hunger; extreme thirst, with violent craving for cold water; violent craving for salt; slow, jerky, taking a long time to say what is slowly going through their minds, evidently making strong efforts to get their thoughts into words, yet never fairly succeeding; unable to differentiate the finer shades of meaning; cannot sing; cannot learn to play an instrument; cannot really reason out the fact that two and two are four. These conditions are perfectly covered and produced by Nat-m.

You will have patients suffering from the poison of Argent-nit., topically applied, either during an ulcerative sore throat, a case of gonorrhœa treated by injections, uterine troubles, or it may have been a chronic ophthalmia—any place upon a delicate mucous membrane that is within reach of the lawless fingers and instruments of a specialist; and it may have left its own cachexia—a poisoning produced by its use in treatment. Again, you will be glad to have known of the *antidotal* record of Nat-m. for Arg-n.

A gonorrhœa having lasted a long time becomes a chronic gleet; gleety, *white*, viscid, mucous discharge, *with burning after urination;* Nat-m.

Marasmus in children; beginning above and moving downward; eats a great deal, ravenously; no assimilation; grows more and more emaciated: Nat-m.

Marasmus in children, with sinking in of the occiput, showing atrophy of the cerebellum, you need not expect to cure. My experience has only shown me these poor little unfortunates among illegitimates, who would so evidently be better out of the world than in it, where there seems to be no place for them.

S. L. G. L.

